# Turning over New Leaves

**Published:** 1888-09-29

**Authors:** 


### Turning Over New Leaves.

[Books for Review should be sent to The Editor, The Lodge, Porchester Square, W.]

### The Intestinal Diseases of Childhood.*

Dr. Jacobi's book is decidedly entertaining, and that is saying a good deal for a medical work. It is also full of recent information and sound reasoning. A great deal of ground is covered in a moderate space, which cannot be affirmed of many medical treatises. The book deals with the intestinal diseases of children, and indeed with the intestines of children whether they be diseased or not. It includes the physiology, hygiene, pathology, and therapeutics of infantile stomachs and bowels—a sufficiently large and decidedly important range of subjects. It cannot be said that children's ailments have received that degree of attention either at medical or non-medical hands which they have deserved. That is perhaps because children are powerless, and are entirely at the mercy of parents, guardians, and doctors. Parents and guardians know practically nothing about the matter, and so the unhappy infant is left, without power of protest, in the hands of whatever doctor may happen to be called in. It is a curious fact, and one which can only be explained on a very unsatisfactory hypothesis, that parents will employ medical men for their children whom they will not trust to attend themselves. It is quite common to hear people say, "I have Dr. Beardless for the children, but I call in Dr. Greyhairs when there is anything the matter with myself." This is shameful. Children are perfectly helpless, and therefore should always have the most skilled and conscientious medical attendance it is possible to procure.

Among other things Dr. Jacobi treats at length the topic of infant feeding—one of the most important and also one of the most neglected of medical questions. On artificial infant foods he lays a most unsparing hand. He dwells with just anger on the fact that the composition of most of these foods is absolutely unknown. Notwithstanding this, fashionable physicians by the score may be found who will laud these unknown compounds to the skies. It is pointed out that milk and the different cereals may be obtained perfectly pure, and that it is possible, by a little trouble, so to make use of these that it shall always be known exactly what a child is taking; exactly, therefore, what makes it thrive on the one hand, and what produces indigestion, pain, and injury on the other. This protest against the use and multiplication of artificial foods of unknown composition is exceedingly well-timed, and demands the most thoughtful consideration on the part of both doctors and parents. The book is small, and, as we have said, is in many places highly entertaining. It ought to have a large sale both within and beyond the pale of the medical profession.

### Canal Boats and Boatmen.†

We have here a narrative of the ways and customs, the habits of thought and action, of an unknown race, a tribe of people of whom, though they live in England at the present day, the average Englishman knows less than he does of the ancient Romans. The canal boatmen are a people apart, keeping and handing down from generation to generation their own traditions of thought and conduct, and coming in contact with the life of other sections of the community only on the dubious meeting-ground of the public-house. The canal boat is something more than a means of transit for coal and bricks and manure: it is the home, the kingdom of a race of practical savages, nearly as ignorant, brutal, and superstitious as any African tribe. In the tiny cabins children are born and families brought up, crowded together in a manner that shames the record of the worst city tenements. They grow up to be cursed, over-worked, and ill-treated by parents who are cruel simply from utter deadness of soul, from lack of sufficient imagination to give the amount of work a child can do; and when they have developed to manhood and womanhood they in their turn become parents as besotted and cruel as those they themselves knew. The children of this tribe of outcasts from our civilization recognize in a father a creature who overworks them when he is sober and curses them when he is drunk, in a mother one who starves them when she is sober and beats them when she is drunk: that there are such beings as parents who do not drink and who are kind to their children is a thing they cannot conceive. They have no God, but they have a devil—the "buggut," who lives in the dark, slimy-walled tunnels, where the water gurgles threateningly, like a monster licking his lips, as the heavy boat is "legged through" by the older boys. The process of "legging through" is pushing the boat along these slimy walls by the vigorous action of strong, bare feet. Sometimes a foot slips, and the boy falls into the black, stagnant water, lost beyond hope of rescue; then the "buggut" is supposed to have pulled him overboard, and his father breathes a curse or two at losing the lad's help, and no one troubles more about the matter. In such utter deadness and darkness the life of the canal people went on till one man strove, through endless disappointment and discouragement, to raise them to a higher and more human level. To him—George Smith, of Coalville—this book is dedicated, and it may be looked upon as to a considerable extent a record of his work. The coffee-room founded by Mr. Bentley, the saving of children from starvation and cruelty, and bringing them up under decent conditions, the gradual instruction and guidance in religion and morality, told here as part of a tale, are actual facts to which we are indebted in the first place to Mr. Smith. Others have followed in his steps, and by degrees—necessarily slow in dealing with such a nomadic population—are wiping away this blot on our civilization. This narrative of gradual redemption as exemplified in the young bargee, Ezra Strong-in-the-arm, and his sister, forms the most interesting part of "Life in the Cut," together with the picture it affords of the ways and thoughts of a section of society which it is to be hoped will soon disappear from the land, absorbed into higher forms of life. But apart from this, the story is a pretty enough one of patient, self-denying love that wins its prize at last, while the trial of Ezra for the murder of Lord Corsham, the heroine's unworthy husband, supplies enough of a tragic element. The author is, however, over-fond of quotations, and his style is sometimes defaced by a striving after fine writing. But, in spite of these defects, "Life in the Cut" is a book worth re[illegible]ding.

* "The Intestinal Diseases of Childhood." By A. Ja obi, M.D. Detroit, Michigan: George L. Davis.

† "Life in the Cut." By Amos Reade. London: Swan Sonnenschein, Lowry, and Co.

